# Evaluation of CT features for differentiating consolidation pattern of pulmonary MALT lymphoma from pneumonic-type lung adenocarcinoma

**DOI:** 10.3389/fonc.2023.1234291

**Published:** 2023-09-01

**Authors:** Congsong Dong, Peng Xia, Wenli Qiu, Zhenyu Dai, Zhongqiu Wang

**Affiliations:** ^1^ Department of Radiology, The Six Affiliated Hospital of Nantong University (Yancheng Third People’s Hospital), Yancheng, China; ^2^ Department of Radiology, Wuxi Traditional Chinese Medicine (TCM) Hospital Affiliated to Nanjing University of Chinese Medicine, Wuxi, China; ^3^ Department of Radiology, Affiliated Hospital of Nanjing University of Chinese Medicine, Nanjing, China

**Keywords:** consolidation pattern of pulmonary mucosa-associated lymphoid tissue, pneumonic-type lung adenocarcinoma, misdiagnosed, computed tomography, differential diagnosis

## Abstract

**Purpose:**

In clinical practice, the consolidation pattern of pulmonary mucosa-associated lymphoid tissue (C-MALT) was often misdiagnosed as pneumonic-type lung adenocarcinoma (P-LADC). However, the mainstay of treatment and prognosis of these two diseases are different. The purpose of this study was to distinguish C-MALT from P-LADC by pre-treatment chest computed tomography (CT) features.

**Patients and methods:**

A total of 31 patients with C-MALT (15 men and 16 women; mean age, 61.1 ± 11.2 years) and 58 patients with P-LADC (34 men and 24 women; mean age, 68.6 ± 7.4 years) confirmed by pathology who underwent contrast-enhanced chest CT were retrospectively enrolled from September 2014 to February 2023. Detailed clinical and CT characteristics of the two groups were evaluated. Logistic regression analysis was used to assess the effectiveness of statistically significant variables in distinguishing C-MALT from P-LADC.

**Results:**

The average age of C-MALT was younger than P-LADC patients (p<0.001). With regard to CT features, bronchiectasis within the consolidation was more common in the C-MALT group than the P-LADC group [83.87% (26 of 31) vs 20.69% (12 of 58), p<0.001]; whereas lymph nodes enlargement [75.86% (44 of 58) vs 9.68% (3 of 31), p<0.001] and pleural effusion [43.10% (25of 58) vs 19.35% (6 of 31), p=0.025] were more frequently observed in the P-LADC group than C-MALT group. The predictors with p<0.05 (age, bronchiectasis, lymph node enlargement, and pleural effusion) were used to construct a logistic regression model in discriminating C-MALT from P-LADC, the area under curve (AUC), positive predictive value (PPV), negative predictive value (NPV), specificity, sensitivity, and accuracy were 0.9555, 86.67%, 91.53%, 83.87%, 93.10%, and 89.89%, respectively.

**Conclusion:**

C-MALT and P-LADC have differential clinical and CT features. An adequate understanding of these different characteristics can contribute to the early accurate diagnosis of C-MALT and provide an appropriate therapeutic strategy.

## Introduction

Primary pulmonary lymphoma (PPL) is an extranodal lymphoma involving the bronchi and/or lung parenchyma, and there is no history of extrapulmonary lymphoma at initial diagnosis or the subsequent 3 months (1). PPL is a rare neoplasm, accounting for 0.4% of all lymphomas, 0.5% of the primary pulmonary malignancies, and 3.6% of extranodal lymphomas. Mucosal-associated lymphoid tissue (MALT) lymphoma, also known as extranodal marginal zone B-cell lymphoma, can arise from any mucosal site and is the most common type of PPL, accounting for 70% to 90% (2). Among them, the lung is the most commonly involved organ, especially the bronchus. Some pulmonary MALT lymphoma patients may have respiratory symptoms such as cough, chest pain, mild dyspnea, and hemoptysis (3). However, these symptoms are non-specific and approximately half of the patients are asymptomatic. Pulmonary MALT lymphoma remains a clinical diagnostic challenge, and currently, definitive diagnosis still relies on histological confirmation.

In recent years, with the improvement of image anatomical resolution, shortening of scanning time, and application of multi-planar reconstruction technology, computed tomography (CT) has become a powerful tool in the evaluation of pulmonary lesions and has been widely used (4). The CT manifestations of pulmonary MALT lymphoma are diversiform (5). On imaging, there are four main types of pulmonary MALT lymphoma: consolidation pattern, nodular mass pattern, ground-glass pattern, and diffuse interstitial pattern (6). Among them, consolidation is the most common pattern manifested as polygon-based parenchymal consolidation involving the lobes/segments of the lung (7). Lung cancer is the main cause of cancer incidence rate and mortality worldwide, and lung adenocarcinoma (LADC) is the most common histological type of lung cancer. Due to the overlap of clinical symptoms and radiological features, the consolidation pattern of MALT lymphoma (C-MALT) can be misdiagnosed as pneumonic-type lung adenocarcinoma (P-LADC) (8). Usually, MALT is indolent and progresses more slowly than LADC, patients with MALT lymphomas have a better prognosis than LADC. For most C-MALT, the mainstay of treatment is chemotherapy and immunotherapy (9, 10); however surgery is the first choice of treatment for P-LADC (11). Therefore, timely and accurate differential diagnosis of P-LADC from C-MALT is crucial for the formulation of appropriate treatment to improve the prognosis of patients.

Although several studies had been conducted to develop characteristic clinical, pathological, and imaging perspectives of pulmonary MALT lymphoma, and imaging perspectives (12), few reports have focused on its differential diagnosis from lung adenocarcinoma. Thus, the aim of the study was to evaluate whether C-MALT can be distinguished from P-LADC on the basis of clinical characteristics and CT imaging manifestations. We hope to increase clinical understanding of C-MALT through this study, improve treatment effectiveness, and reduce the risk of misdiagnosis of this disease.

## Materials and methods

The retrospective study protocol was approved by the ethics committee of the Affiliated Hospital of Nanjing University of Chinese Medicine, and the patient informed consent was waived because of the retrospective, observational, and anonymous nature of this research.

### Patients

**Figure 1:**
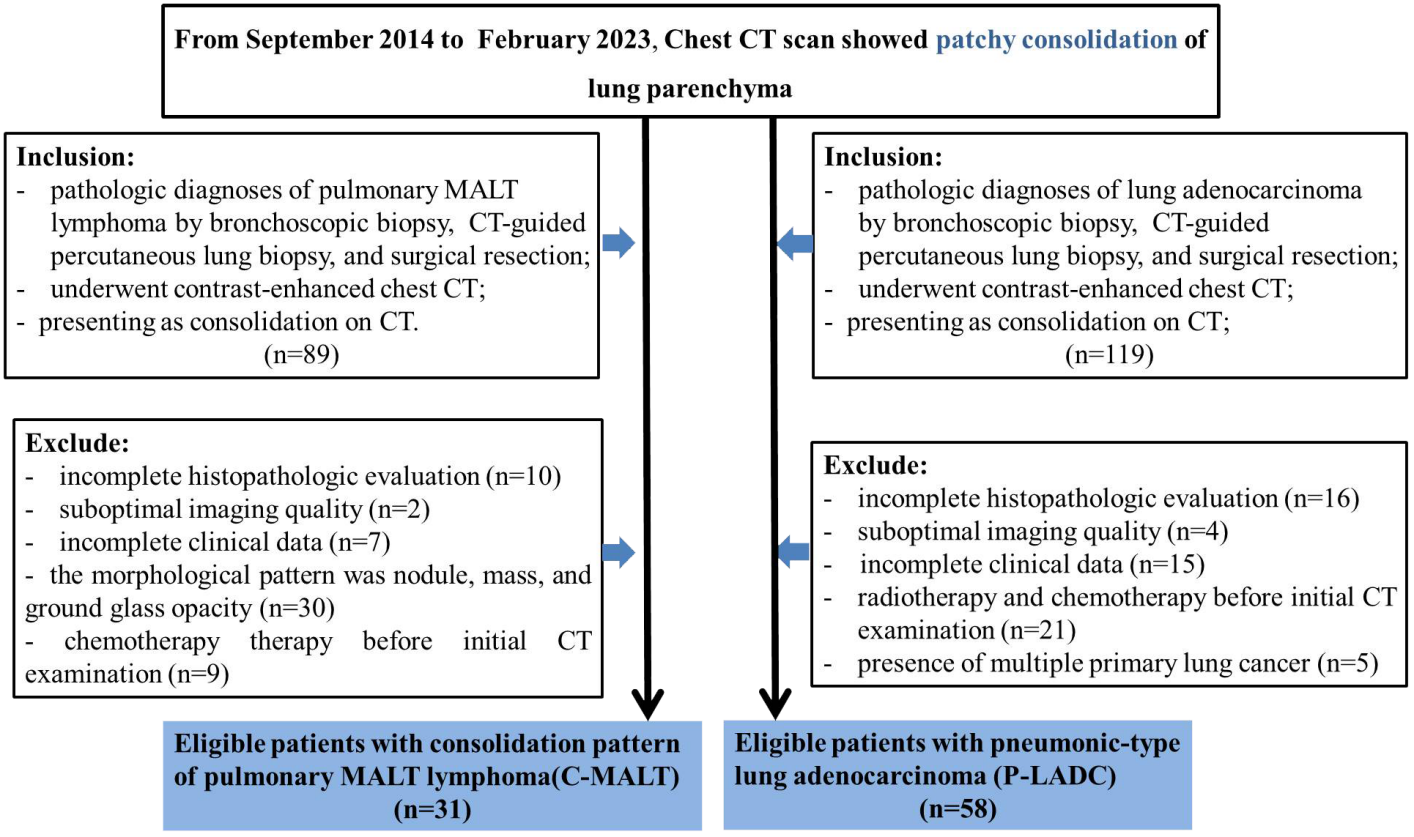
Patients selection flow diagram. CT, computed tomography; C-MALT, consolidation pattern of pulmonary mucosa-associated lymphoid tissue; P-LADC, pneumonic-type lung adenocarcinoma.

From September 2014 to February 2023, we collected the data of 89 patients with pulmonary MALT lymphoma who met the following inclusion criteria: (1) pathologically proven pulmonary MALT lymphoma; (2) contrast-enhanced chest CT scan; (3) CT results showed patchy consolidation of lung parenchyma. The exclusion criteria were as follows: (1) incomplete histopathologic evaluation (n=10); (2) suboptimal imaging quality (n=2); (3) incomplete clinical data (n=7); (4) the morphological pattern was nodule, mass, and ground glass opacity (n=30); (5) chemotherapy therapy before initial CT examination (n=9). As a result, a total of 31 patients were enrolled in the C-MALT group (**Figure 1**).

Similarly, from September 2014 to February 2023, we collected the data of 119 patients with lung adenocarcinoma who met the following inclusion criteria: (1) pathologically proven lung adenocarcinoma; (2) contrast-enhanced chest CT scan; (3) CT results showed patchy consolidation of lung parenchyma. The exclusion criteria were as follows: (1) incomplete histopathologic evaluation (n=16); (2)suboptimal imaging quality (n=4); (3) incomplete clinical data (n=15); (4) radiotherapy and chemotherapy before initial CT examination (n=21); (5) presence of multiple primary lung cancer (n=5). As a result, a total of 58 patients were enrolled in the P-LADC group (**Figure 1**).

### CT protocols

Chest CT scanning was performed using Brilliance 64 (Phillips Healthcare) or Light Speed 64VCT (GE Healthcare) following the standardized protocol. CT scanning was performed from the thoracic inlet to the level of bilateral adrenal glands in the supine position. Unenhanced CT scanning was first performed, and imaging parameters were as follows: tube voltage, 120 kVp; tube current, 100–250 mA; beam pitch, 0.516–0.98; slice thickness, 5 mm; and slice interval, 5 mm. For contrast-enhanced examination, the patients were injected with 80-110 mL (at a dosage of 1.5 mL/kg of body weight) nonionic contrast medium (iohexol 300 mg/mL; Omnipaque, GE Healthcare) + 30 mL physiological saline using a dual high-pressure injector (Stellant, Medrad, Indianola, USA) at a flow rate of 3.0 mL/s. Images of the arterial phase and delayed phase were obtained at 30 and 120 s after the start of injection, respectively. Subsequently, CT images were reconstructed into 0.625 or 1 mm slice thickness with a sharp reconstruction algorithm. All images were performed with both lung window (window width, 1500 HU; window level, −700 HU) and mediastinal window (window width, 375HU; window level, 50 HU).

### Image analysis

If the patient had multiple lesions, the single largest lesion was used for analysis, and if the patient had undergone at least two CT scans, the first CT scan was analyzed. All the chest CT images were retrospectively and independently evaluated by two experienced radiologists (WQ and CD) with more than 10 years of experience in chest imaging, both of them were blinded to the clinical, laboratory, and histopathological information. Any disagreements in the assessment were resolved by discussion until a consensus opinion was obtained. The following CT imaging features were evaluated: lung lesion distribution (unilateral or bilateral), location (right upper lobe, right middle lobe, right lower lobe, left upper lobe, or left lower lobe), size (product of the maximum diameter and the shortest diameter of the lesion in the lung window setting), margin (well-defined or ill-defined), calcification (present or absent), air bronchogram sign (present or absent), and bronchiectasis(present or absent) within the consolidation, bulging of interlobar fissure (present or absent), hilar or mediastinal lymph nodes enlargement (shorter diameter >1cm, present or absent), and pleural effusion (present or absent). CT attenuation values were obtained by using a post-processing workstation (Syngo.via, Siemens Force, Germany)

### Statistical analyses

Statistical analyses were performed using the software package SPSS version 22.0 for Windows (IBM, Chicago, IL, USA). The categorical variables were presented as numbers and percentages and compared by Chi-square test, while the continuous variables were presented as mean ± standard deviation and compared by Student’s t-test. A two-tailed *p*<0.05 was considered statistically significant. Using logistic regression analysis to assess the effectiveness of statistically significant variables in distinguishing between C-MALT and P-LADC, different predictive models were constructed. Construct Receiver operating characteristic (ROC) curve, and the area under curve (AUC), positive predictive value (PPV), negative predictive value (NPV), specificity, sensitivity, and accuracy were used to measure their diagnostic performance.

## Results

### Study population

A total of 31 patients with C-MALT and 58 patients with P-LADC were finically included for analysis (**Figure 1**). Among the 31 patients with C-MALT (15 men and 16 women; mean age, 61.1 ± 11.2 years; age range, 32–78 years), 12 were diagnosed by bronchoscopic biopsy, 9 were diagnosed by CT-guided percutaneous lung biopsy, and 10 were diagnosed by surgical resection. Similarly, of the 58 patients with L-PLADC (34 men and 24 women; mean age, 68.6 ± 7.4 years; age range,46–81 years), 15 were diagnosed by bronchoscopic biopsy, 14 were diagnosed by CT-guided percutaneous lung biopsy, and 30 were diagnosed by surgical resection.

### Comparison of clinical characteristics between C-MALT and P-LADC

The clinical characteristics of patients with C-MALT and 58 P-LADC are described in **Table 1**. The average age of patients with C-MALT was younger than that of P-LADC [61.1 ± 11.2 (32–78) vs 68.6 ± 7.4 (46–81)] with a p-value less than 0.001. However, no significant difference was observed in gender, respiratory symptoms, and elevation of white blood cell count between the two groups with a p-value of 0.355, 0.764, and 0.836.

**Table 1:** The clinical characteristics between C-MALT and P-LADC.

Characteristics	C-MALT (n=31)	P-LADC (n=58)	*p*-value				
Age (years)			<0.001				
Mean ± SD	61.1 ± 11.2	68.6 ± 7.4					
(Range)	(32-78)	(46-81)					
Gender, n (%)			0.355				
Male	15 (48.39%)	34 (58.62%)					
Female	16 (51.61%)	24 (41.38%)					
Symptoms, n (%)	15 (48.39%)	30 (51.72%)	0.764				
Respiratory symptoms	15 (48.39%)	30 (51.72%)					
Asymptomatic	16 (51.61%)	28 (48.29%)					
Elevation of white blood cell count			0.836				
Increased	7 (22.58%	12 (20.69%)					
Normal	24 (77.42%)	46 (79.31%)					

### Comparison of CT characteristics between C-MALT and P-LADC

**Figure 2:**
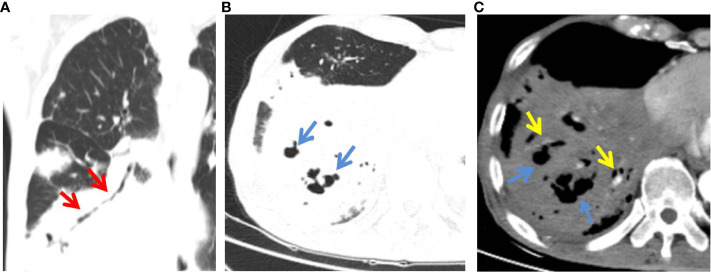
C-MALT lymphoma in a 58-year-old woman with right back pain for 2 weeks. **(A)** Lung-window HRCT multi-planar reconstruction showed a consolidation with air bronchogram (red arrow) in the right lower lobe. **(B)** There was cystic bronchiectasis (blue arrow) within the lesion. **(C)** Mediastinum-window showed consolidation with cystic bronchiectasis (blue arrow) and angiogram sign (yellow arrow). HRCT, high-resolution computed tomography; C-MALT, consolidation pattern of pulmonary mucosa-associated lymphoid tissue.

**Figure 3:**
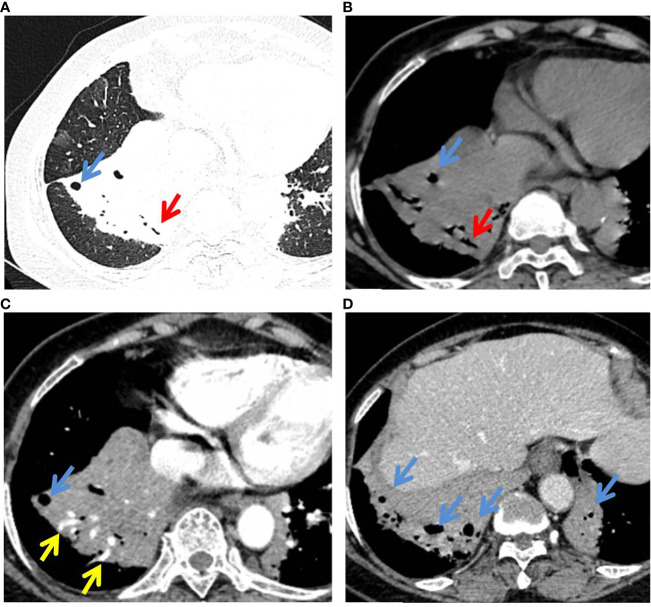
P-MALT lymphoma in a 66-year-old with a cough for 14 days. **(A)** Axial CT image of the lung window indicated multiple consolidations with air bronchogram (red arrow) and cystic bronchiectasis (blue arrow) in the left lower lobe and right lower lobe. **(B–D)** Mediastinum-window in the plain scan, arterial phase, and venous phase showed air bronchogram (red arrow), cystic bronchiectasis (blue arrow), and angiogram sign (yellow arrow) within the lesions. CT, computed tomography; C-MALT, consolidation pattern of pulmonary mucosa-associated lymphoid tissue.

**Figure 4:**
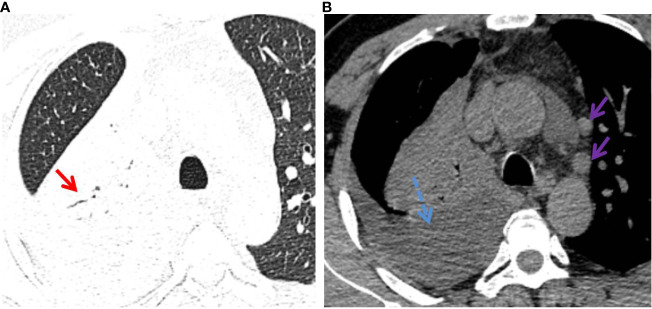
P-LADC in a 70-year-old man without symptoms. **(A)** Axial HRCT of the lung window showed a consolidation with air bronchogram (red arrow) in the right upper lobe. **(B)** Mediastinum window showed right pleural effusion (blue dashed arrow) and multiple enlarged lymph nodes in the mediastinum (purple arrow). HRCT, high-resolution computed tomography; P-LADC, pneumonic-type lung adenocarcinoma.

**Figure 5:**
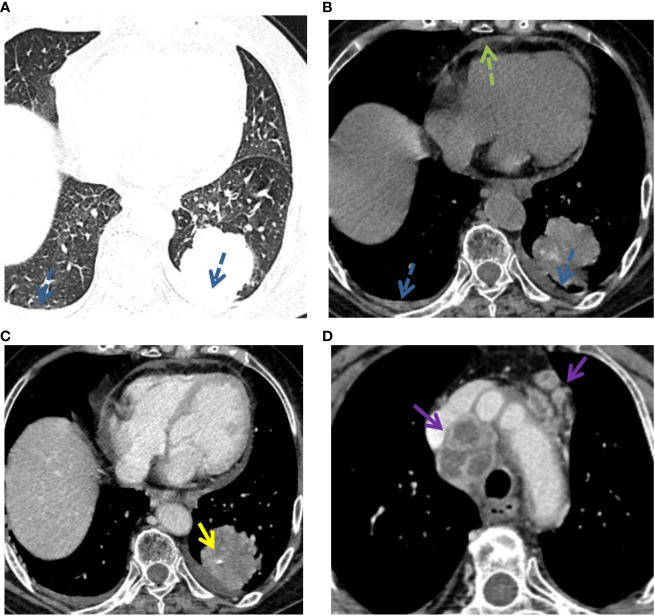
P-LADC in a 77-year-old man with cough and hemoptysis for 12 days. **(A)** Axial lung-window HRCT showed a localized consolidation in the left lower lobe and bilateral pleural effusion (blue dashed arrow). **(B)** Mediastinum window showed bilateral pleural effusion (blue dashed arrow) and pericardial effusion (green dashed arrow). Arterial phase indicated **(C)** an angiogram sign (yellow arrow) within the lesion and **(D)** multiple enlarged lymph nodes with necrosis in the mediastinum (purple arrow). HRCT, high-resolution computed tomography; P-LADC, pneumonic-type lung adenocarcinoma.

The detailed CT characteristics of C-MALT and P-LADC groups are summarized in **Table 2**. A total of 48 lesions were found in 31 C-MALT patients, including 24 unilateral lesions and 7 bilateral lesions. A total of 78 lesions were found in 58 P-LADC patients, including 46 unilateral lesions and 12 bilateral lesions (*p*=0.836). The lesions were randomly distributed, and there was no statistical difference in the distribution of lesions in each lung lobe between the two groups (*p*=0.74). There were no significant statistical differences were observed in lesion size, margin, CT attenuation value, air bronchogram sign, angiogram sign, calcification, and interlobular fissure bulging between C-MALT (**Figures 2**, **3**) and P-LADC (**Figures 4**, **5**) groups (all *p* > 0.05). Bronchiectasis within the lesion was more common in the C-MALT group than the P-LADC group [83.87% (26 of 31) vs 20.69% (12 of 58), p<0.001] (**Figures 2**, **3**), and cystic bronchiectasis was observed in 16 C-MALT patients; whereas lymph nodes enlargement [75.86% (44 of 58) vs 9.68% (3 of 31), p<0.001] and pleural effusion [43.10% (25of 58) vs 19.35% (6 of 31), p=0.025] were more frequently observed in the P-LADC group than C-MALT group (**Figures 4**, **5**).

**Table 2:** CT imaging features between C-MALT and P-LADC.

CT Features	C-MALT (n=31)	P-LADC (n=58)	*p*-value
Distribution			
Laterality			0.836
Unilateral	24 (77.42%)	46 (79.31%)	
Bilateral	7 (22.58%)	12 (20.69%)	
Location			0.746
Right upper lobe	14 (29.17%)	17 (21.79%)	
Right middle lobe	4 (8.33%)	8 (10.26%)	
Right lower lobe	9 (18.75%)	14 (17.95%)	
Left upper lobe	13 (27.08%)	19 (24.36%)	
Left lower lobe	8 (16.67%)	20 (25.64%)	
Lesion size (cm^2^) (range)	40.84 ± 15.72 (8.65-110.09)	45.61 ± 19.88 (9.37-123.21)	0.251
Margin			0.428
Well-defined	16 (51.61%)	35 (60.34%)	
Ill-defined	15 (48.39%)	23 (39.66%)	
CT attenuation value (HU) (range)	39.63 ± 9.73 (20.8-55.0)	37.01 ± 9.80 (16.3-58.3)	0.232
Air bronchogram sign			0.156
Present	29 (93.55%)	48 (82.76%)	
Absent	2 (6.45%)	10 (17.24%)	
Angiogram sign			0.740
Present	26 (83.87%)	47 (81.03%)	
Absent	5 (16.14%)	11 (18.97%)	
Bronchiectasia			<0.001
Present	26 (83.87%)	12 (20.69%)	
Absent	5 (16.13%)	46 (79.31%)	
Calcification			0.717
Present	2 (6.45%)	5 (8.62%)	
Absent	29 (93.55%)	53 (91.38%)	
Interlobular fissure bulging			0.248
Present	14 (45.16%)	19 (32.76%)	
Absent	17 (54.84%)	39 (67.24%)	
Lymph node enlargement			<0.001
Present	3 (9.68%)	44 (75.86%)	
Absent	28 (90.32%)	14 (24.14%)	
Pleural effusion			0.025
Present	6 (19.35%)	25 (43.10%)	
Absent	25 (80.65%)	33 (56.90%)	

### Assessment of diagnostic performance of models

**Figure 6:**
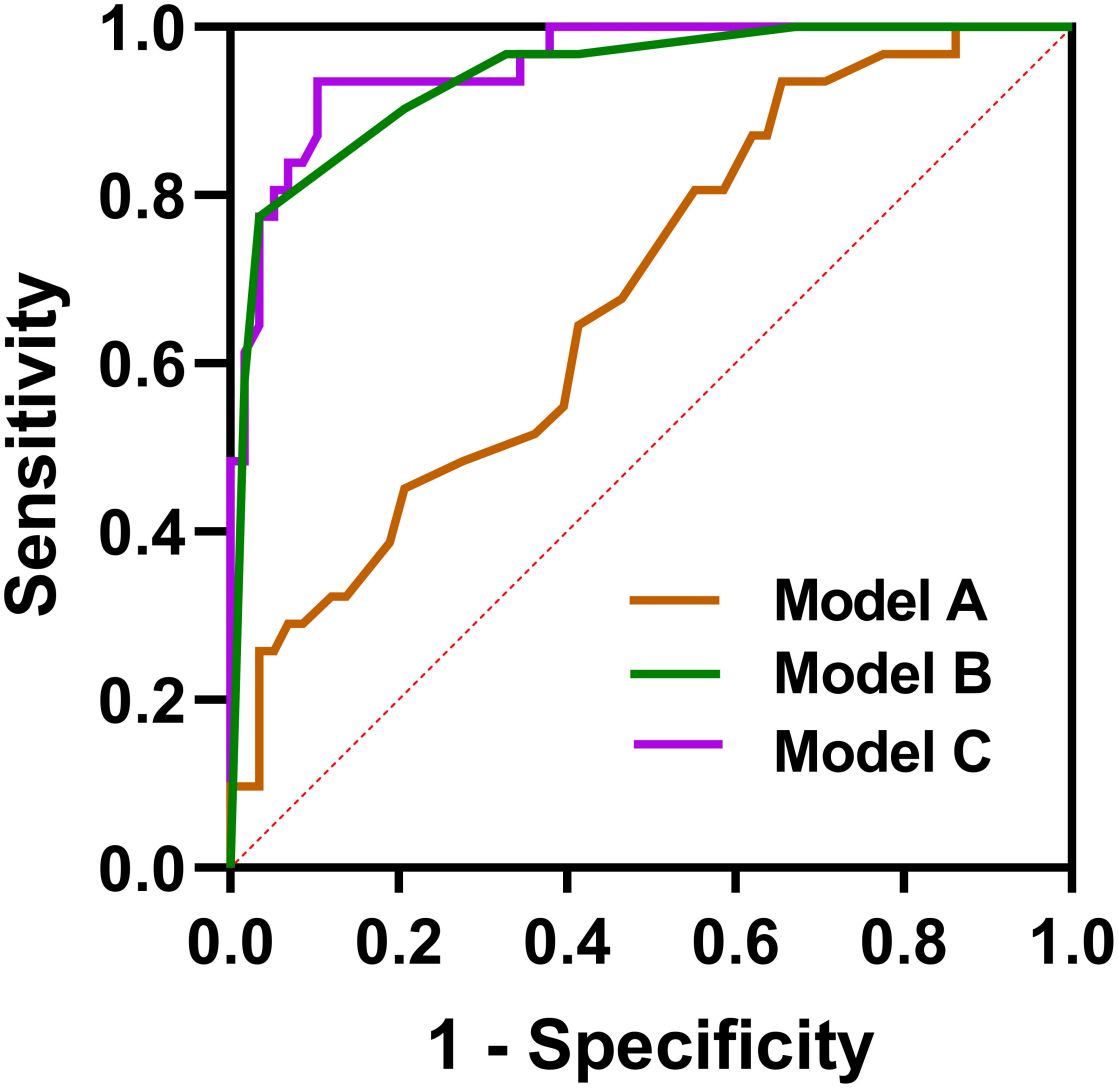
Receiver operating characteristic curves. Model A: age, Model B, bronchiectasis + lymph node enlargement + pleural effusion; Model C, age + bronchiectasis + lymph node enlargement + pleural effusion.

We constructed three logistic regression models using predictors with p <0.05 in univariate analysis, including age, bronchiectasis, lymph node enlargement, and pleural effusion, to distinguish C-MALT from P-LADC: model A (clinical feature-based model), age; model B (CT characteristic-based model), bronchiectasis+lymph node enlargement+pleural effusion; and model C (model A+ model B), age + bronchiectasis+lymph node enlargement+pleural effusion. The diagnostic performances of the three models in discriminating C-MALT from P-LADC were shown in **Table 3**, and the ROC curves were shown in **Figure 6**. The AUC value, PPV, NPV, specificity, sensitivity, and accuracy of model A were 0.6841, 64.29%, 70.67%, 29.03%, 91.38%, and 69.66%, respectively; the AUC value, PPV, NPV, specificity, sensitivity, and accuracy of model B was 0.9397, 92.31%, 88.89%, 77.42%, 96.55%, and 89.89%, respectively; the AUC value, PPV, NPV, specificity, sensitivity, and accuracy of model C was 0.9555, 86.67%, 91.53%, 83.87%, 93.10%, and 89.89%, respectively.

**Table 3:** Diagnostic performance of age, bronchiectasis, lymph node enlargement, and pleural effusion in differentiating C-MALT from P-LADC.

	AUC (95% CI)	PPV (%)	NPV (%)	SPE (%)	SEN (%)	Accuracy (%)
Model A	0.6841	64.29	70.67	29.03	91.38	69.66
Model B	0.9397	92.31	88.89	77.42	96.55	89.89
Model C	0.9555	86.67	91.53	83.87	93.10	89.89

Model A, age; Model B, bronchiectasis + lymph node enlargement + pleural effusion; Model C, age + bronchiectasis + lymph node enlargement + pleural effusion.

C-MALT, consolidation pattern of pulmonary mucosa-associated lymphoid tissue; P-LADC, pneumonic-type lung adenocarcinoma; AUC, area under the curve; CI, confidence interval; PPV, positive predictive value; NPV, negative predictive value; SPE, specificity; SEN, sensitivity.

## Discussion and conclusion

The treatment of C-MALT and P-LADC is different, misdiagnosis of C-MALT as P-LADC may lead to overtreatment, and misdiagnosis of P-LADC as C-MALT may lead to delayed surgical treatment (13, 14). Therefore, it is crucial to distinguish between C-MALT and P-LADC before the treatment. In clinical practice, clinical and radiological features are considered valuable tools for distinguishing them (15). Although several researchers have given attention to assessing the clinical, pathological, and imaging characteristics of C-MALT, few reports have focused on its differential diagnosis from P-LADC, and non-invasive distinguishing C-MALT from P-LADC in clinical practice is a challenge. Therefore, in the present study, we compared the clinical characteristics and CT imaging features of 31 patients with C-MALT and 58 patients with P-LADC, and established a logistic regression model to distinguish them. The results demonstrated that age, bronchiectasis within the consolidation, lymph node enlargement, and pleural effusion showed great potential in distinguishing C-MALT from P-LADC.

C-MALT patients mostly appear at about the age of 60, but young patients have also been reported (16), while P-LADC patients are mostly elderly and relatively older (17). In the study, we found the average age of patients of C-MALT was younger than that of P-LADC, which was consistent with previous studies. Some scholars indicated that nearly half of MALT lymphoma patients are asymptomatic, while some with clinical symptoms may present with respiratory symptoms such as cough, chest pain, mild dyspnea, and hemoptysis, similar to lung adenocarcinoma (18, 19). Consistent with previous studies, we found no significant difference in clinical symptoms between C-MALT and P-LADC patients(20). Furthermore, no significant difference was observed in gender and elevation of white blood cell count between the two groups.

The primary CT manifestation of C-MALT is large and lamellar consolidation involving lobe/segment, which is often misdiagnosed as P-LADC in clinical practice (21). In this study, we found that most C-MALT and P-LADC lesions were randomly distributed regardless of transverse and longitudinal distribution, with no obvious trend of central distribution, which was similar to the previous study (22). Although there was no significant difference was observed in air bronchogram signs between C-MALT and P-LADC groups, the pathological mechanisms were different. In C-MALT, tumors originate from the pulmonary interstitium and grow along or infiltrate the interstitial lung and bronchial submucosal epithelium, mainly destroying the pulmonary interstitium and bronchial wall is inviolable, therefore, residual bronchial shadows are often seen within the lesion (23). In the early stage of P-LADC, the tumor cells rarely obliterate the underlying pulmonary architecture, including the bronchi, resulting in the bronchial lumen staying smooth. As the tumor grows, due to the tumor invasion and desmoplastic reaction, the air bronchogram gradually disappears in P-LADC (24). The angiogram sign is a CT feature of pulmonary MALT lymphoma, but it is nonspecific as it is also observed in other diseases, such as lobar pneumonia, organizing pneumonia, pulmonary atelectasis, and lung adenocarcinoma, representing vascular wall destruction is absent (25, 26). Herein, there was no significant difference in angiogram signs between C-MALT and P-LADC groups. In accordance with previous studies, we also found there was no significant difference was observed between C-MALT and P-LADC in terms of lesion size, margin, CT attenuation value, calcification, and interlobular fissure bulging with p>0.05 (27).

Our results indicated that bronchiectasis within the consolidation was more frequently detected in the C-MALT group than P-LADC group (83.87% vs 20.69%, p<0.001). Hence, we supposed that bronchiectasis (especially cystic bronchiectasis) within the consolidation, as one of the relatively characteristic CT manifestations of C-MALT, helps to distinguish C-MALT from P-LADC (5). It is different from the permanent bronchial wall alterations observed in traditional bronchiectasis; there is no tumor necrosis or bronchial wall destruction in the dilated bronchus in C-MALT (28). The pathological basis of bronchial or bronchiolar dilation might result from the collapse and destruction of parenchyma adjacent to bronchus secondary to infiltration and proliferation of lymphoid tissue (22).

We found lymph node enlargement (75.86% vs 9.68%, *p*<0.001) and pleural effusion (43.10% vs 19.35%, *p*=0.025) were more frequently observed in the P-LADC group than C-MALT group. Consistent with other studies, a defining feature of MALT was the absence of significant mediastinal or hilar adenopathy. In the opinion of most researchers, MALT is essentially extranodal lymphomas according to the diagnostic criteria, rarely involving hilar and mediastinal lymph nodes (29). In addition, pleural invasion, as indicated by pleural effusion was less frequent in C-MALT than P-LADC.

In this study, we also constructed a logistic regression model using predictors with p<0.05 including age, bronchiectasis, lymph node enlargement, and pleural effusion, to distinguish C-MALT from P-LADC. Our data indicated that the model had good diagnostic efficacy with high AUC of 0.9555, 86.67% PPV, 91.53% NPV, specificity of 83.87%, sensitivity of 93.10%, and accuracy of 89.89%, suggesting that these features could assist in distinguishing C-MALT from P-LADC. Based on this result, we concluded that mastering these differential features was helpful for the accurate diagnosis of C-MALT, not only reducing the unnecessary surgical resection rate of C-MALT but also avoiding P-LADC delaying the optimal surgical timing (30).

There are several limitations that should be considered. First, this study was retrospective in nature, which might lead to selection bias. Second, given that the majority of consolidation pattern of lung cancer is found in adenocarcinomas, our studies were limited to adenocarcinomas, and other pathological types were not included. Further other pathological types of lung cancers should be investigated. Third, although the number of patients with C-MALT enrolled in the study was higher than in most previous studies, the number was still relatively small due to the rarity of C-MALT and our strict inclusion criteria. Therefore, prospective multicenter larger-cohort research may be needed to further confirm our results in future studies.

In conclusion, our findings demonstrated that C-MALT and P-LADC have differential clinical characteristics and CT features. An adequate understanding of these different characteristics can contribute to providing diagnostic clues for C-MALT and lead the clinician to make appropriate therapeutic strategies.

## Data availability statement

The original contributions presented in the study are included in the article/supplementary material. Further inquiries can be directed to the corresponding author.

## Ethics statement

The retrospective study protocol was approved by the ethics committee of the Affiliated Hospital of Nanjing University of Chinese Medicine. The studies were conducted in accordance with the local legislation and institutional requirements. The ethics committee/institutional review board waived the requirement of written informed consent for participation from the participants or the participants’ legal guardians/next of kin because of the retrospective, observational, and anonymous nature of this research. Written informed consent was not obtained from the individual(s) for the publication of any potentially identifiable images or data included in this article because of the retrospective, observational, and anonymous nature of this research.

## Author contributions

Design of experiment: ZD and ZW; acquire the clinical data: CD, PX, and WQ; image analysis: CD, WQ, and ZW; paper drafting: CD and WQ; data analysis: PX; paper correction: WQ. All authors contributed to the article and approved the submitted version.
